# Recalibrating differential gene expression by genetic dosage variance prioritizes functionally relevant genes

**DOI:** 10.1101/gr.280360.124

**Published:** 2025-10

**Authors:** Philipp Rentzsch, Aaron Kollotzek, Kaushik Ram Ganapathy, Pejman Mohammadi, Tuuli Lappalainen

**Affiliations:** 1 Science for Life Laboratory, Department of Gene Technology, KTH Royal Institute of Technology, 17165 Solna, Sweden;; 2 Department of Integrative Structural and Computational Biology, Scripps Research Institute, La Jolla, California 92037, USA;; 3 Center for Immunity and Immunotherapies, Seattle Children's Research Institute, Seattle, Washington 98101, USA;; 4 Department of Pediatrics, University of Washington School of Medicine, Seattle, Washington 98105, USA;; 5 Department of Genome Science, University of Washington, Seattle, Washington 98195, USA;; 6 New York Genome Center, New York, New York 10013, USA;; 7 Department of Systems Biology, Columbia University, New York, New York 10032, USA

## Abstract

Differential expression (DE) analysis is a widely used method for identifying genes that are functionally relevant for an observed phenotype or biological response. However, typical DE analysis includes selection of genes based on a threshold of fold change in expression under the implicit assumption that all genes are equally sensitive to dosage changes of their transcripts. This tends to favor highly variable genes over more constrained genes where even small changes in expression may be biologically relevant. To address this limitation, we have developed a method to recalibrate each gene's DE fold change based on genetic expression variance observed in the human population. The newly established metric ranks statistically differentially expressed genes, not by nominal change of expression, but by relative change in comparison to natural dosage variation for each gene. We apply our method to RNA sequencing data sets from in vitro stimulus response and neuropsychiatric disease experiments. Compared to the standard approach, our method adjusts the bias in discovery toward highly variable genes and enriches for pathways and biological processes related to metabolic and regulatory activity, indicating a prioritization of functionally relevant driver genes. Tissue-specific recalibration increases detection of known disease-relevant processes. Altogether, our method provides a novel view on DE and contributes toward bridging the existing gap between statistical and biological significance. We believe that this approach will simplify the identification of disease-causing molecular processes and enhance the discovery of therapeutic targets.

Since the advent of cDNA microarrays (Schena et al. 1995), differential gene expression profiling has been used to examine the characteristic gene regulatory changes of a specific phenotype, disease state, or perturbation. Statistical tests are used to determine whether the observed differences in expression of each single gene between groups of samples are statistically significantly differentially expressed (DE) over a chosen statistical threshold. Over the years, many different bioinformatics methods to test DE have been developed, adapting rigorous statistical tests to the particularities and assumptions of DNA microarrays and later RNA-seq. Some of the currently most popular methods for differential gene expression analysis are DESeq2 (Love et al. 2014), edgeR (Robinson et al. 2010), and limma-voom (Ritchie et al. 2015). These tools are designed to work with the count-based nature of RNA-seq data and incorporate normalization procedures to account for differences in sequencing depth and RNA composition across samples. Like most statistical tests, DE methods have been shown to sometimes produce false positives, particularly when analyzing RNA-seq data sets of large sample sizes. To address this issue, several different approaches of multiple testing correction have been developed (Benjamini and Hochberg 1995; Ge et al. 2009; Ignatiadis et al. 2016) and are applied in many of the popular methods.

Whereas DE testing quantifies the statistical significance of DE, it is agnostic to its *biological relevance*, that is, whether the detected change in gene expression meaningfully reflects or contributes to changes in cellular functions. This means that a test's *P*-value does not carry any inherent meaning (Greenland et al. 2016; Wasserstein and Lazar 2016), and well-powered DE studies can result in hundreds, if not thousands, of DE genes. As a consequence, the actual expression fold change—the ratio of the expression levels between the two sample groups—is often considered an important secondary parameter (Cui and Churchill 2003; McCarthy and Smyth 2009; Zhang and Cao 2009; Jung et al. 2011; Harrison et al. 2019). Many DE studies apply an arbitrarily chosen minimum fold change threshold and inspect only statistically significant genes surpassing this cutoff. This makes sense insofar as the magnitude of an expression change is obviously relevant (Naqvi et al. 2023) and, for a single gene, bigger expression changes tend to matter more than smaller ones. However, the absolute fold change in itself is of limited relevance when comparing changes between different genes because different genes have different levels of dosage constraint (Rice and McLysaght 2017). This means that a measured change in expression in one gene may be comparable to biological noise and may fall within the spectrum of natural variation in the population. However, a deviation of the same magnitude in another gene can be highly unusual and lead to immediate cellular consequences, either by affecting other genes or by directly altering the phenotype. Such a change could hence be described as not only statistically but also biologically significant. Prior work shows that genes with constrained expression are enriched for drivers of cellular processes and disease (Lek et al. 2016; Rice and McLysaght 2017; Mohammadi et al. 2019; Karczewski et al. 2020; Collins et al. 2022; Dong et al. 2023). It follows that the subsequent difference in gene responsiveness makes nominal fold change a poor proxy for biological relevance, an effect that may contribute to the systematic differences between gene expression changes and associations between genetic variants and traits (Mostafavi et al. 2023).

The best studied quantity that describes how tolerant genes are to dosage variation is haploinsufficiency, the gene's intolerance to heterozygous deletion or loss-of-function variants. A related concept of triplosensitivity refers to intolerance to duplication. Beyond these large changes that affect one entire copy of a gene, especially noncoding genetic variants affect the expression of nearby genes. For many genes, as recently exemplified by the gene encoding the transcription factor SOX9 (Naqvi et al. 2023), these regulatory changes are associated with differences in phenotype and disease risk (Albert and Kruglyak 2015). To our knowledge, there exist currently no assays that measure the viable dosage range of mRNA expression for each gene in a genome. However, it has been shown that haploinsufficiency of genes aligns well with the amount of purifying selection in the genome (Lek et al. 2016; Karczewski et al. 2020; Collins et al. 2022). Indeed, depletion of genetic variation in populations has been a powerful genome-wide approach for detecting molecular variation that is not tolerated by natural selection.

In this study, we have developed a novel calibration method to measure DE by scaling standard expression changes with the population genetic dosage variation estimate V^G^, a metric which we had previously introduced (Mohammadi et al. 2019). This calibration approach was tested and validated in multiple previously published data sets, and the resulting recalibrated gene expression fold changes were evaluated for their ability to improve the interpretability of DE analyses across diverse data sets and tissues. The objective of this method is to provide a more accurate framework for identifying genes that play a pivotal role in regulating cellular processes and disease mechanisms.

## Results

### Variance in gene expression as a fold change recalibration metric

The amount of population variability in expression is different for each gene. Whereas some of the measured variability is due to technical factors, physiological differences, or external influences from the environment, some of it is due to genetic differences between individuals. To infer the component of natural variation that is due to genetic *cis*-regulatory differences, we previously introduced ANEVA, a method for assessing the gene expression distribution based on allelic expression (AE) of heterozygotic SNPs or eQTLs (Mohammadi et al. 2019). Recently, we have further extended this principle to use allelic expression over entire haplotypes (ANEVA-h), improving robustness and data coverage (K Ganapathy, M Broly, S Silverstein, et al., in prep.; https://zenodo.org/records/15226575). Applying ANEVA-h to GTEx v8, an RNA-seq data set from more than 800 individuals, we derived an estimate of gene expression variance (V^G^) for nearly all human genes. We refer to these estimates as V^G^_H_. Because both expression and variability depend on body tissue and cell type, V^G^_H_ is initially calculated separately for 50 different tissues in GTEx. An overall V^G^_H_ value is then calculated as the weighted harmonic mean across tissues.

**Figure 1.**
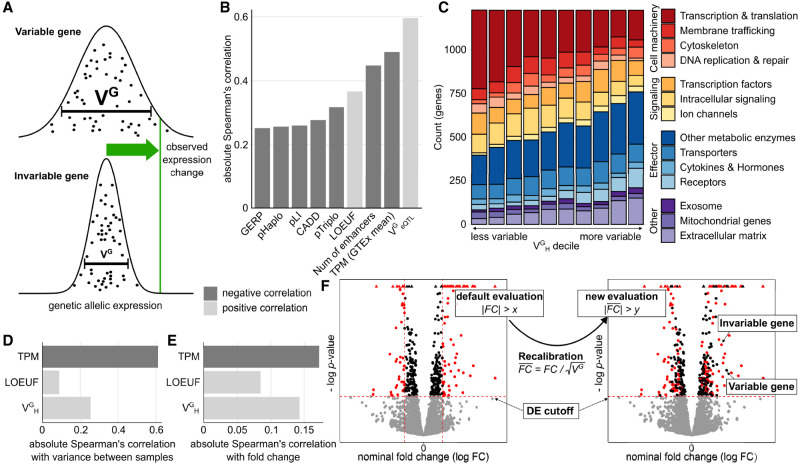
Fold change recalibration using V^G^. (*A*) Depending on variability of a gene, an observed fold change may be within the natural variation that is observed in the general population. V^G^ is an estimate of this population variance. (*B*) Correlation of V^G^_H_ with other gene metrics. Negative correlations are colored dark gray; positive correlations are light gray. (*C*) Distribution of functional categories in genes split by V^G^_H_ decile. Some functional categories like transcription- and translation-associated genes were enriched for small V^G^_H_ estimates, and others, like receptors, were enriched for high V^G^_H_ estimates. (*D*) Correlation of gene metrics with experimental gene expression variance. Data sourced from Alasoo et al. (2018) studying the effects of IFNG treatment on 81 human macrophage samples. (*E*) Correlation of gene metrics with absolute gene expression changes in the IFNG experiment. (*F*) Concept of gene expression recalibration using V^G^. As a result of this process, genes are prioritized not by nominal log fold change (*FC*) but by recalibrated log fold change ($\bar{FC}$).

As V^G^ is based on the allelic fold change (Mohammadi et al. 2017) of genetic regulatory effects, its unit is analogous to log fold changes used in DE analyses. The magnitude of V^G^ describes the range in which observed gene expression changes fall within the population (Fig. 1A). Large values imply greater variability and thus a wider range of expression levels in the population. V^G^ hence serves as a parameter to classify an expression change relative to the respective gene's mean, that is, where a sample with the observed change would fall within the natural (population) range.

In our previous publication (Mohammadi et al. 2019), we showed that AE-based V^G^ (V^G^_AE_) of a gene is correlated with selective constraint. Known haploinsufficient genes have lower V^G^_AE_ on average, and V^G^_AE_ is correlated with other constraint metrics such as conservation scores or the probability of being loss-of-function intolerant (pLI). Here, we measured the correlation of our most recent, haplotype derived V^G^_H_ estimates to additional gene constraint metrics (Fig. 1B). One of the most highly correlated metrics (*ρ*_Spearman's_ = 0.36) is the loss of function tolerance metric LOUEF (Karczewski et al. 2020). Most interesting are the haploinsufficiency and triplosensitivity metrics pHaplo and pTriplo (Collins et al. 2022). Whereas V^G^_H_ is less correlated with both metrics than, for example, LOEUF, we found that, in contrast to the latter, V^G^_H_ is slightly more correlated with pTriplo (*ρ*_Spearman's_ = −0.31) than pHaplo (*ρ*_Spearman's_ = −0.25), indicating its potential in capturing sensitivity to both up- and downregulation. The high negative correlation with the number of enhancers (*ρ*_Spearman's_ = −0.46) suggests that the genetic population variance in expression is linked to buffering at the molecular level (Wang and Goldstein 2020).

In addition to metrics of constraint, we found that V^G^ estimates are associated with gene function. As described for a simpler GTEx-derived “allelic Fold Change” metric (Dong et al. 2023), genes annotated as part of the central cellular processes, particularly those involved in transcription and translation, are enriched among genes with low V^G^_H_. In contrast, effector proteins such as receptors, as well as extracellular matrix proteins, are more likely to have a high V^G^_H_ (Fig. 1C).

We next investigated how V^G^ is correlated to gene expression variance in experimental data. To this end, we analyzed a data set of 81 human macrophage samples that were treated with IFNG (Alasoo et al. 2018). As expected due to the well-documented mean-variance relationship, expression level (transcripts per million [TPM]) was highly correlated with interindividual variance between control samples, but also V^G^_H_ and LOEUF were correlated to variance (Fig. 1D), which is not unexpected for different metrics of population variance. More notably, the expression fold change upon IFNG stimulus was also correlated with V^G^_H_ (*ρ*_Spearman's_ = 0.143, 95%-confidence interval [CI_95_] = [0.125;0.161]) (Fig. 1E), significantly more so than LOEUF (*ρ*_Spearman's_ = 0.085, CI_95_ = [0.066;0.103]) and within the range of TPM (*ρ*_Spearman's_ = −0.172, CI_95_ = [−0.190;−0.155]). Whereas DE significance testing accounts for the variance in the studied data set, the correlation between fold change and V^G^_H_ indicates that genes that are more likely to be noticeable outliers in a DE study also have a higher population variance in general.

The idea behind the original ANEVA method was to have a test that evaluates whether the genetic effect in a gene in one particular sample is an outlier compared to the genetic variance in the population. Conversely, here, we propose to use the derived variance metric V^G^ to rescale nongenetic gene expression differences between two groups in order to compare expression changes across different genes on the same scale, relative to the population variance in each. Whereas the above findings provide support for using V^G^ to rescale gene expression fold changes, the main motivation for V^G^ as a metric itself is unit equivalence. Unlike scores such as LOEUF, GERP, CADD, or pLI, where there is no direct relationship between score value and variability, a gene with a V^G^ of 0.02 is twice as variable as a gene with a V^G^ of 0.01. We used this property to recalibrate the experimental log fold change by scaling it by the average change in expression. This average change in expression is the standard deviation, which is the square root of the variance. Thus, with this approach, which we call recalibration, we standardize the observed fold change for each gene with the standard deviation of the genetically regulated gene expression given by the square root of V^G^ (Fig. 1F). To distinguish between pre- and postrecalibration, we refer to log fold changes obtained from an experiment as “nominal” and fold changes after rescaling as “recalibrated.” Recalibration is an additional analysis step after significance testing, and it changes the relative order of genes assigned as significant based on standard DE testing. This means that small changes in expression for some genes may be considered relatively more significant than the same change would be for other genes.

### Recalibration shifts the analysis focus

**Figure 2.**
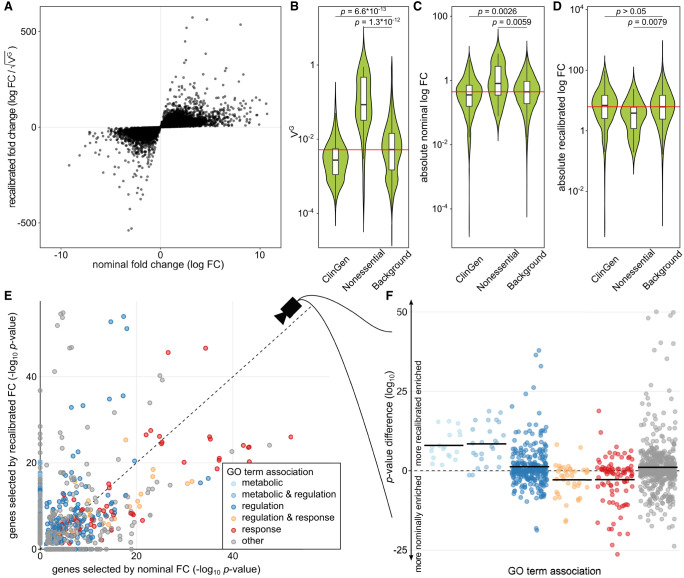
Fold change recalibration of a DE experiment of IFNG treatment. (*A*) Nominal compared to recalibrated fold changes of individual genes from Alasoo et al. (2018). (*B*–*D*) Impact of recalibration on genes from three different gene sets: ClinGen, Nonessential, and all other genes (Background). Part *B* displays the distribution of V^G^_H_ per set; part *C*, the absolute log fold changes before recalibration (nominal); and part *D*, the recalibrated absolute log fold changes. Each violin represents the total distribution of values, with boxes in the violin indicating 25% and 75% quantiles and the median illustrated as a black line. Whiskers are 1.5 times the interquartile range. The red line denotes the median over all genes. Significance labels are the result of a two-sided comparison with Mann–Whitney *U* test of the two groups. (*E*) Effect on GO term enrichment: adjusted *P*-values of GO term enrichment from 2000 genes selected by nominal compared to recalibrated FC. GO-terms are colored based on string matching (association). The dashed *center* line marks the same enrichment in both sets. GO terms *above* the line are more enriched after the recalibration, and terms *below* the line are more enriched before. (*F*) Different view on the change in GO term enrichment *P*-values grouped by associations. Colored dots are GO terms; the colored vertical lines are the log-mean per association. Terms containing the words “regulation” (blue) and “metabolic” (light blue) are, on average, more enriched after recalibration, whereas terms containing the word “response” (red) are, on average, more enriched before recalibration.

To assess the effect of a recalibration with V^G^, we used the previously introduced data set of 81 human macrophage samples stimulated with IFNG. After processing the samples and selecting DE genes with a strict false discovery rate of less than 0.001, the data set yields 20,402 DE genes (out of 29,438 in the data set). Of these, 5573 genes also have an absolute FC greater than 1. The data set hence serves as an excellent illustration of the need for selection criteria beyond statistical significance to identify the most relevant genes for further analysis. To compute recalibrated fold changes, we restricted further analysis to the 22,650 genes in the data set for which V^G^_H_ estimates have been calculated. Of these, 17,494 were DE and 4586 DE genes had an aFC greater than 1. The recalibrated fold changes were significantly correlated with the nominal fold changes (Pearson's correlation of absolute values: 0.52) (Fig. 2A).

To examine the effect of the recalibration, we first turned to sets of well-characterized genes. Known haploinsufficient genes from ClinGen on median had lower than average V^G^_H_ estimates, whereas those of nonessential genes were generally higher than average (Fig. 2B). Nonessential genes had higher absolute fold changes upon IFNG stimulus than ClinGen and background genes, indicating that a standard DE analysis might easily focus on these functionally irrelevant genes (Fig. 2C). The recalibration results in an adjustment of the fold change ranges for the different gene sets (Fig. 2D), with their respective distributions no longer being significantly different.

Using the 2000 most DE genes by nominal and recalibrated absolute FC, respectively, we observed a shift in GO term enrichments (Fig. 2E; Supplemental Table S1). In particular, terms matching the string “response,” like “immune response” (GO:0006955) and “response to other organism” (GO:0051707), were less enriched, whereas terms matching “regulation” like “regulation of biological process” (GO:0050789), or “metabolic” like “metabolic process” (GO:0008152), were more enriched among the genes selected after recalibration (Fig. 2F). This effect was robust to the number of genes analyzed (Supplemental Fig. S1) or when the gene set enrichment was calculated based on gene ranks (GSEA) (Supplemental Fig. S2). However, we note that many GO terms represent broad categories of genes rather than specific pathways, and GO term enrichments are not independent of each other as most genes are associated with many different GO terms. We analyzed the extent of sharing by clustering the GO terms by the genes associated with each term, finding that the two main clusters including GO terms whose names contain the word “response” are, on average, less enriched after recalibration, whereas 10 out of 12 other GO term clusters where multiple terms match the word “regulation” are more enriched after recalibration (Supplemental Fig. S3). However, it should be noted that, whereas the differences in enrichment can be large and only 991 genes are in the top 2000 in both lists, the change in the number of genes per GO term is small (Supplemental Fig. S4). We found the same enrichment of GO terms matching “regulation” and “metabolic” when performing the same analysis on experiments of macrophages perturbed with *Salmonella* and *Salmonella*+IFNG from the same experimental data source (Supplemental Fig. S5). We also found the same trend when recalibrating using eQTL-based V^G^ estimates (Supplemental Fig. S6). All these results indicate that nominal fold change captures *response* to stimuli, whereas recalibration enriches for *regulators* of cellular response.

Next, we wanted to analyze whether this pattern holds for a DE study of a more limited size than the well-powered Alasoo et al. (2018) data. To this end, we analyzed the data from Findley et al. (2021) with multiple in vitro stimuli of three different cell lines in six biological replicates, focusing on the response to metal ions. A known key mechanism in this response is a family of proteins, metallothioneins, that have the ability to bind metal ions, providing protection against metal toxicity (Sekovanić et al. 2020). In the stimulus experiments of copper and zinc ion solutions, the top 10 DE genes based on classical fold change include five and seven of the total of nine metallothionein genes, respectively (Supplemental Figs. S7A, S8A), and the associated GO terms “cellular response of copper/zinc ion” (GO:0071280) are significantly GO enriched. Recalibration reduces the metallothionein gene ranking and GO enrichment (Supplemental Figs. S7B, S8B), as the V^G^_H_ values of the metallothionein genes are in the top quartile of all genes.

Altogether, these analyses show how the choice of DE approach depends on the goals of the analysis: the standard approach readily picks up genes that respond to the given stimulus. However, recalibration provides a gradual adjustment to DE gene rankings, which may help to deprioritize highly variable responder genes to highlight molecular regulators and drivers of cellular response.

### Tissue-specific expression fold change recalibration

**Figure 3.**
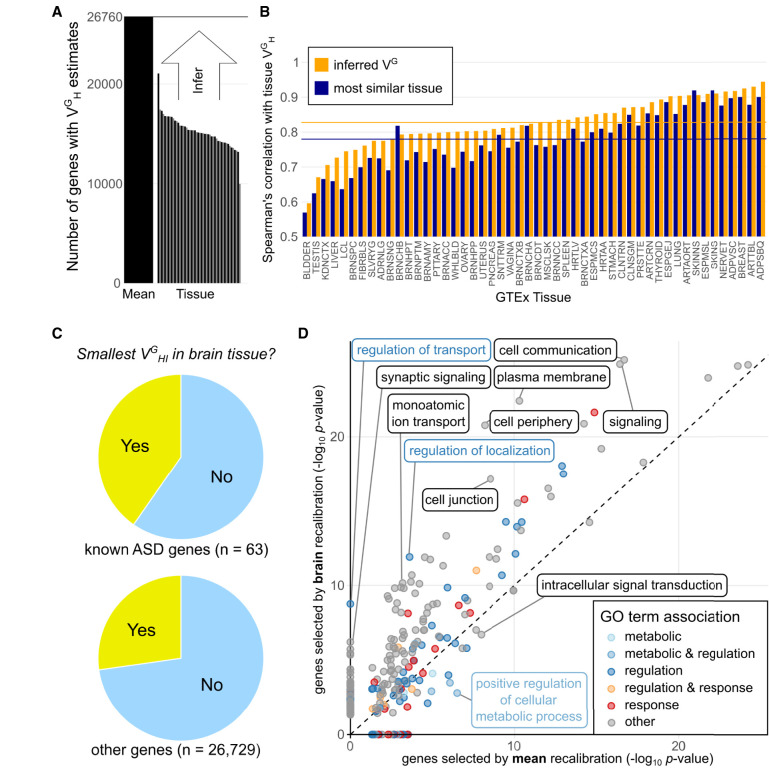
Tissue-specific recalibration. (*A*) Number of genes V^G^_H_ is estimated for, both as the tissue agnostic mean and per tissue. (*B*) Per tissue correlation of tissue V^G^_H_ estimates with the inferred V^G^_I_ estimate, compared to correlation with the most highly correlated tissue. Horizontal lines are medians for all tissues. (*C*) Comparison in which tissue the V^G^_HI_ of a gene is lowest. Compared to all other genes, known ASD genes are significantly more likely to have the lowest V^G^_HI_ in one of the GTEx brain tissues. (*D*) Impact on GO term enrichment of using tissue-specific V^G^_HI_ estimates (GTEx tissue Brain Frontal Cortex) compared to mean V^G^_H_ for recalibration in a DE experiment by PsychENCODE comparing ASD patients and controls. These results indicate that high-coverage tissue-specific metrics of regulatory constraint from the new haplotype-based V^G^ estimates in GTEx increase the detection of biologically meaningful expression signatures in disease.

The analyses presented so far use mean V^G^_H_ values across tissues, which does not account for tissue-specific differences in variability. The logical alternative is to use tissue-specific V^G^ estimates of the closest tissue available. However, whereas the mean V^G^_H_ estimates cover 26,760 genes, only between 9992 and 21,051 genes had estimates in each of the 50 tissues (Fig. 3A). Based on the previous finding that estimates of V^G^ between tissues correlate with tissue expression (Mohammadi et al. 2019), we developed an inference method of estimating V^G^ based on the expression in other, correlated tissues (Supplemental Fig. S9; Methods). We refer to the generated V^G^ estimates as inferred (V^G^_I_). V^G^_I_ is, for most tissues, more correlated to V^G^_H_ (median 0.82) (Fig. 3B) than the most strongly correlated other GTEx tissue (median 0.77), with the exception being tissues like the two GTEx skin tissues that are much more similar to each other than any other tissue. Due to the simplicity of the method, it allows us to generate V^G^_I_ estimates for all 26,760 genes in all 50 GTEx tissues. To recalibrate as many genes as accurately as possible, we combined V^G^_H,TISSUE_ and V^G^_I,TISSUE_ into V^G^_HI,TISSUE_, with V^G^_H_ taking precedence.

Tissue-specific V^G^_HI_ estimates allow for experiment-specific recalibration in order to highlight genes under constraint in that tissue. For example, we find that a significantly increased fraction of previously known autism spectrum disorder (ASD) genes (An et al. 2018) has the lowest V^G^_HI_ in one of the 14 GTEx brain tissues (binomial test *P*-value of 5.2 × 10^−4^) (Fig. 3C). This indicates that V^G^, unlike genetics-based scores, can capture tissue-specific regulatory constraint. To explore this further, we used the psychENCODE data set (Gandal et al. 2018), which studied ASD, schizophrenia (SCZ), and bipolar disorder (BD) across hundreds of RNA-seq samples primarily from the prefrontal cortex.

The complete psychENCODE data set measured the expression of 25,772 genes. For recalibration, we used the V^G^_HI_ estimates for 19,164 genes of the GTEx Brain - Frontal Cortex tissue. Of the 1611 statistically significant DE genes in ASD versus controls, 1444 have V^G^ estimates and can be recalibrated. First, we replicated the general pattern of recalibration enrichment for regulatory GO categories compared to nominal fold change ranking, analogous to those in the IFNG stimulus data (Supplemental Fig. S10; Supplemental Table S2). Importantly, GO term enrichments among the top 700 genes after recalibration with tissue-specific V^G^ estimates compared to mean V^G^ showed improved results (Fig. 3D), detecting enrichments for GO terms such as “regulation of transport” (GO:0051049) and “synaptic signaling” (GO:0099536), which were not significantly enriched when selecting genes based on recalibration with mean V^G^ estimates. Synaptic signaling is well-characterized as a key process in ASD (Jiang et al. 2022), whereas regulation of transport contains genes which modulate the transport of various molecules across cellular membranes. Using the tissue-specific V^G^ for recalibration, we observed similar increased enrichments of neural activity-linked GO terms like cell communication and signal transduction for both SCZ (Supplemental Fig. S11) and BD (Supplemental Fig. S12).

## Discussion

Here, we have introduced a novel approach for one of the most common analyses in computational biology: ranking of DE genes. Using a genetic regulatory variance metric for recalibration—as opposed to metrics relying on transcriptome or genetic data alone—has many advantages. Firstly, it provides a robust and generalizable estimate of biological constraint on expression that is not confounded by intermingled technical and biological noise in transcriptome studies. Most constraint scores, particularly those based on genetic variation, do not capture tissue specificity, which we have shown here to further increase the enrichment of likely regulatory pathways. Furthermore, using *genetic* variance to better interpret environmental or other *nongenetic* differences brings together two typically distinct areas of biological enquiry. Whereas a simpler method (Starr et al. 2023) has been proposed for assessing whether an observed expression change is different from the natural variability, V^G^ is based on allelic fold changes (Mohammadi et al. 2017) that are measured in the same unit as DE fold change. Thus, this is the first method to compare different genes from an experiment on the same, biologically interpretable scale.

We have shown that recalibration deprioritizes highly variable genes and removes the bias of DE results, easily highlighting nonessential genes with high population variation. Reranking of DE genes led to increased enrichment of molecular processes related to regulation and correctly identified key driver processes in neuropsychiatric traits, although the lack of broadly applicable gold-standard annotations of gene function beyond GO categories limits the resolution of these insights. Indeed, whereas we show that recalibration produces robust patterns for gene sets, users are encouraged to interpret results from individual genes with some caution. Nevertheless, our results indicate that our approach has the potential to identify phenotypic drivers, guiding downstream analyses toward genes and processes that are biologically causal. These would represent potential targets for interventions. Conversely, the standard DE approach is sensitive in picking up genes that reflect the response to the signal of interest. Whereas such genes do not necessarily drive downstream processes, they may be informative—for example, as biomarkers of external stimulus. Thus, the standard and recalibrated DE analyses highlight different types of biology, both of which may be of interest. An additional interesting group of genes that our current approach is not particularly well suited to highlight include genes where high genetic and environmental variability contributes to adaptation to different environments (Kelly et al. 2012). However, the ability to focus the downstream analysis to phenotypic drivers and deprioritize highly variable genes that merely reflect the response will be valuable in many applications.

The approach presented here has some limitations, many of these derived from the input data for V^G^ estimates. Despite our innovations in deriving V^G^ estimates for a substantially increased number of genes, especially in tissue-specific analyses, many genes still lack an empirically derived V^G^ estimate, although this can be successfully alleviated with the presented inference methods. Missing genes are generally lowly expressed, which is the reason why allele-specific data (and similarly eQTLs) could not be determined and that low-expressed genes are generally more variable and thus negatively affected by recalibration. Thus, missing genes are likely to be negatively correlated to functional importance, unlike for some genetics-based scores (Lek et al. 2016). Furthermore, allelic analysis is highly sensitive to potential clonality in cell lines, making some data sets unsuitable for V^G^ estimation.

Another key aspect is the context specificity of V^G^. Our previous findings indicate that genetic background has only a minor effect on gene expression variability (Mohammadi et al. 2019), although it is unclear if there are more subtle differences and exceptions that cannot be captured with current data sets. Our results both highlight the importance of tissue-specific V^G^ and show a high correlation of closely related tissues, likely deriving from some strong genetic *cis*-regulatory effects being shared between tissues (The GTEx Consortium 2020). This is likely to extend to different cell states, where gene-environment (G×E) interactions—that is, response eQTLs—might create situations where V^G^ calculated on baseline data will not fully capture genetic variability under a different cell state. However, major G×E effects affect a relatively small number of genes (Manuck and McCaffery 2014; Kim-Hellmuth et al. 2017; Wang et al. 2019). Thus, recalibration can be done with tissue, cell type, or cell state proxies, but growing gene expression data sets will further improve the quantity and quality of V^G^ estimates that closely match the differential expression data, which is likely to improve the quality of the recalibration.

Our results demonstrate that the current V^G^ scores are of sufficient quality for our straightforward recalibration to yield biologically meaningful signals. This evidence will hopefully inspire future incorporation of V^G^ noise estimates in the fold change recalibration step and potentially even into DE significance testing itself to account for general population variance. In such analyses, accurate matching of the cell type and sometimes even the population background between the experiment and V^G^ values is likely to become an even higher priority.

Recalibration changes the unit of differential expression from molecular fold change to how many standard deviations an expression change is from the population mean. Neither approach has a natural threshold of biological significance, and we prefer the ranking approach where top N genes are inspected. The ultimate way to identify molecular changes that are biologically meaningful are experimentally intensive, but an exciting future direction, will be to map the complex and often nonlinear relationships of molecular expression changes, downstream phenotypic consequences, and population variance (Naqvi et al. 2023; Domingo et al. 2024).

In summary, we have introduced a new approach for prioritizing potential regulatory drivers in DE analyses. This provides yet another demonstration of the value of population-scale RNA-seq data for enhancing biological interpretability of broadly used study designs and enabling new discoveries in the future.

## Methods

### Genetic variance in gene expression

V^G^ is a metric that estimates the genetic variance in expression for genes in the human population, which we introduced in previous publications (Mohammadi et al. 2017, 2019). There, we described approaches based on different data types to calculate V^G^: from allelic expression based on single SNVs (AE), from allelic expression based on haplotype information (H), and from expression quantitative trait loci (eQTL). Briefly, AE-based V^G^ (V^G^_AE_) is derived based on the comparison of allele read counts for each single nucleotide variant (SNV) in the two haplotype copies in a diploid individual. This information is aggregated by SNV frequency over all individuals in the tested population and fitted via ANEVA (analysis of expression variation), a model for population AE data, to infer with V^G^_AE_ the level of genetic expression variability between haplotypes. Haplotype-based V^G^ (V^G^_H_), in contrast, is derived by aggregating read counts across phased haplotype blocks. Here, we are using the haplotype-expression matrices from GTEx v8, that were generated with phASER using WASP corrected RNA-seq data (Castel et al. 2020). This aggregation increases the total read counts and enhances the number of genes with detectable allelic expression. The aggregated data are then fitted with a simplified version of the ANEVA model, replacing the aggregation of probabilites over multiple SNVs with a single binomial-logit-normal probability estimator to infer V^G^_H_ (K Ganapathy, M Broly, S Silverstein, et al., in prep.; https://zenodo.org/records/15226575). In contrast, V^G^_eQTL_ is generated from the most significant eQTL of a gene as an aggregate of effect size and allele frequency. All approaches of generating V^G^ have been applied to RNA-seq samples from the GTEx project, generating separate estimates for each GTEx tissue. A mean V^G^ has been calculated as a weighted harmonic mean over all tissue-specific V^G^ estimates, using expression per tissue, measured in TPM, as weight.

V^G^_H_ estimates used in this study were generated on data from GTEx v8 and were reused from K Ganapathy, M Broly, S Silverstein, et al., in prep. (https://zenodo.org/records/15226575). V^G^_eQTL_ estimates are based on GTEx v7 and were reused from the Mohammadi et al. (2019) publication. Genes for which no V^G^ has been estimated were excluded from the analyses. All V^G^_H_ estimates are provided in the supplement (Supplemental Table S3).

### Recalibrating expression fold change

In order to prioritize biological significance, expression fold change (FC) from DE analysis is standardized relative to the standard deviation of genetically regulated gene expression:

(1) $${\bar{FC}}_{g}=\frac{FC_{g}}{\sqrt{V_{g}}},$$

where *FC*_*g*_ and ${\bar{FC}}_{g}$ are the nominal and recalibrated log fold change of a gene *g*, and *V*_*g*_ is V^G^, the expected variance in gene dosage introduced by genetic variation in the population. An implementation of the process can be found in Supplemental Code S1.

### Other gene metrics

RVIS scores per gene were obtained from Petrovski et al. (2013). ncCADD, ncGERP, and ncRVIS were obtained from Petrovski et al. (2015). The numbers of enhancers and super-enhancers per gene were derived from GeneHancer v5 (Fishilevich et al. 2017). Based on the GFF file with all enhancers from genecards.org, enhancers with a score ≥0.7 and a gene association “link_score” ≥7 were filtered. The final numbers represent the total counts after filtering per gene. The index for tissue-specific expression in GTEx tau (Palmer et al. 2021) was downloaded from genomics.senescence.info/gene_expression/Tau_score.zip. The triplosensitivity and haploinsufficiency scores, pTriplo and pHaplo, respectively, were obtained from the supplemental data of Collins et al. (2022). The same data set was further used to obtain EpiScore (Han et al. 2018). pLI (Lek et al. 2016) and LOEUF (Karczewski et al. 2020) scores were obtained from gnomAD (https://gnomad.broadinstitute.org), using data from the v3 release. Transcripts per million values for each tissue were downloaded from GTEx (https://gtexportal.org/home/), using GTEx version 8. When referring to TPM in the text, we are generally using the mean TPM across all GTEx tissues, which were, in turn, calculated as the median across all individual samples of each tissue.

To account for the different scales of different gene metrics, correlations with V^G^ were calculated as Spearman's correlation, And 95% confidence intervals of correlation were determined using bootstrapping.

The list of nonessential genes was obtained from Zarrei et al. (2015). ClinGen genes were downloaded via the dosage sensitivity curated gene list from ftp.clinicalgenome.org/ClinGen_gene_curation_list_GRCh38.tsv (version from 2023/02/13). Haploinsufficient genes were selected by joining all genes with a haploinsufficiency score of 1, 2, or 3. The background gene set contains all genes with H-based V^G^ values, except for the genes included in one of the two other gene sets. Genes with a TPM of <1 were filtered.

### KEGG gene annotations

The annotation of KEGG functional categories was adapted from Dong et al. (2023). We reused their functional category labels but excluded the category “Domain-containing proteins not elsewhere classified” due to frequent overlaps with other categories. When multiple labels could be applied to a gene, we prioritized the one that appeared least frequently in the list of all genes with V^G^. Genes that were not found in KEGG or that did not match any of the labels were omitted.

### RNA expression data processing

RNA expression data from Alasoo et al. (2018) were downloaded from Zenodo (https://zenodo.org/record/839011 and https://zenodo.org/record/4678936). The data sets contain the results after DE testing with DESeq2 and count data, respectively. DE was determined by selecting genes with an adjusted *P*-value of <0.001. Recalibration was performed using the mean V^G^_H_ values.

The Findley et al. (2021) data sets of different perturbation agents were downloaded from the supplemental data of the publication. The experiments mentioned in the analysis are “15C1” (copper perturbation) and “20C1” (zinc perturbation) of iPSCs from the shallow sequencing data set. The downloaded data sets contain the results of DE testing with DESeq2. DE was determined by selecting genes with a false discovery rate of <0.05. Recalibration was performed using the mean V^G^_H_ values.

The psychENCODE data sets from Gandal et al. (2018) for ASD, SCZ, and BD were downloaded from the supplemental data. The data sets contain the results after DE testing with DESeq2. DE was determined using a false discovery rate cutoff of 0.05. Recalibration was performed using the inference-assisted V^G^_H_ (i.e., V^G^_HI_) estimates for the tissue BRNCTXB (Brain Frontal Cortex BA9).

### Correlation between nominal and recalibrated fold changes

In order to avoid inflation caused by directionality (which does not change), we calculated the correlation between nominal and recalibrated fold changes using the Pearson's method of the absolute values.

### GO term enrichment

Gene Ontology (GO) term enrichment analysis was conducted using the R package g:Profiler2 (Raudvere et al. 2019; Kolberg et al. 2023), using GO terms and gene associations from the Biological Process (BP), Cellular Component (CC), and Molecular Function (MF) GO resources based on Ensembl release 110. Enrichments were calculated based on Ensembl gene identifiers against all genes with determined expression and V^G^ values in the data set. Genes were selected by absolute nominal fold change |*FC*| and absolute recalibrated fold change $\bar{FC}$, respectively. For all enrichment comparisons except the testing of other gene numbers, the top ∼50% (rounded to the nearest 100) of all significant genes were selected. Only genes from the experiment for which V^G^ has been calculated were included as the background set. Enrichment was considered up to an adjusted *P*-value of 0.05. In the comparison of nominal and recalibrated fold changes, nonenriched GO terms were imputed with a *P*-value of 1.

GO term clustering to identify groups of GO terms with a high degree of shared genes was performed with the help of the R package simplifyEnrichment (Gu and Hübschmann 2023) using binary-cut clustering. Clustering had to be performed separately for terms of each GO resource (BP, CC, and MF).

### Gene set enrichment

Gene Set Enrichment Analysis (GSEA) was performed using the R package clusterProfiler (Wu et al. 2021) using FGSEA (Korotkevich et al. 2021) as backend. In contrast to GO term enrichment based on groups, only GO terms and gene association from the GO Biological Process resource were used, and the analysis was performed on all genes (irrespective of statistical significance) for which gene expression and V^G^ are defined. Enrichment was considered up to an adjusted *P*-value of 0.05. Corresponding nonenriched GO terms were imputed with a *P*-value of 1.

### Inferring V^G^ estimates for additional tissues

The implemented inference method for gene V^G^ estimates per tissue comprises a series of subsequent steps, illustrated in Supplemental Figure S9 and added as Supplemental Code S2. The inference is based on the two 26,760 × 50 matrices: *E* for gene expression (measured in transcripts per million) and *V* for V^G^_H_, with 26,760 being |*G*|, the number of genes *G* and 50 being |*T*|, the number of tissues *T*. Note that not all values in *V* are defined, as those are the estimates that are to be inferred. To circumvent issues with null values for very low-expressed genes, $\bar{E}\;$ is defined by adding a pseudocount equal to the smallest expression value larger than 0 that is observed over all genes *G*:

(2) $$\bar{E}=E+\underset{g\in E,t\in T,E_{g,t}\;>\;0}{\min}E_{g,t}.$$

From these data, a model is fitted for each tissue *t* ∈ *T* over all genes *g* ∈ *G*. Accordingly, a linear least-square fit linking $\log(\bar{E})$ and log(*V*) is calculated for each tissue *t*, using the function “lm” in R:

(3) $$\log({\bar{E}}_{t})=\beta_{0,t}+\beta_{1,t}\;*\;\log(V_{t})\;+\epsilon .$$

Tissue V^G^_H_ estimates are then adjusted using the fit to $\hat{V}$, the expression variance that would be expected if the tissue expression was equal to the mean expression over all tissues for that gene:

(4) $${\hat{V}}_{g,t}=V_{g,t}\;*\;{\left(\left(\frac{1}{\left|T\right|}\sum_{r\in T}{\bar{E}}_{g,r}\right)/\;{\bar{E}}_{g,t}\right)}^{\beta_{1,t}}.$$

The degree of similarity between the V^G^_H_ estimates of different tissues *t*_1_ and *t*_2_ is determined using the Spearman's correlation coefficient *ρ* as

(5) $$\rho (t_{1},t_{2})=\mathrm{SpearmansCorrelation}(V_{t_{1}},V_{t_{2}}).$$

For each gene *g* in each tissue *t*, we then define a set *S*_*g*,*t*_ ⊂ *T* consisting of the five tissues *s* ∈ *S*_*g*,*t*_ other than *t* that have the highest degree of similarity *ρ*(*t*, *s*) and for which *V*_*g*,*s*_ is defined. If *V*_*g*_ is defined for less than five tissues, then only those tissues are included. Unadjusted inferred V^G^ estimates $\dot{V}$ are then calculated as a weighted mean of the estimates from the similar tissues, using *ρ* as weight:

(6) $${\dot{V}}_{g,t}=\frac{\sum_{s\in S_{g,t}}\rho (t,s)\cdot {\hat{V}}_{g,t}}{\sum_{s\in S_{g,t}}\rho (t,s)}.$$

As the final step, ${\dot{V}}_{g,t}$ is adjusted to the expression value ${\bar{E}}_{g,t}$ using the fit from Equation 3:

(7) $${\tilde{V}}_{g,t}={\left({\bar{E}}_{g,t}/\left(\frac{1}{\left|T\right|}\sum_{r\in T}{\bar{E}}_{g,r}\right)\right)}^{\beta_{1,t}}\;*\;{\dot{V}}_{g,t}.$$

We refer to $\tilde{V}$ as inferred V^G^ estimates or V^G^_I_. To increase the number of predicted genes, existing tissue estimates of V^G^_H_ were combined with V^G^_I_, with existing scores taking precedence if both existed for a particular gene, in what is called V^G^_HI_. All V^G^_HI_ estimates are available in Supplemental Table S4.

### ASD genes

Previously ASD-associated genes were downloaded from An et al. (2018) using the most stringent list with FDR < 0.1. The tissue with the lowest V^G^ per gene was calculated from V^G^_HI_. GTEx brain tissues are all 13 GTEx tissues starting with “BRN” plus pituitary. Enrichment was determined using a one-sided binomial test.

### Data analysis

All data analysis was done in the R programming language (R Core Team 2021).

## Data access

All V^G^_H_ estimates are provided in Supplemental Table S3. All V^G^_HI_ estimates are provided in Supplemental Table S4. An R package for recalibrating experimental DE data is available at GitHub (https://github.com/LappalainenLab/recalibrate). Version 1.0.0 of the recalibrate package is also provided as Supplemental Code.

## Supplemental Material

Supplement 1

Supplement 2

Supplement 3

Supplement 4

Supplement 5

Supplement 6
