# Supplementary material for: Recalibrating differential gene expression by genetic dosage variance prioritizes functionally relevant genes
Source: Genome Res. 2025 Oct;35(10):2316–25. doi: 10.1101/gr.280360.124 (PMC12487816; doi:10.1101/gr.280360.124)
Supplement: Supplement 6 [file Supplemental_Material.pdf]

SUPPLEMENTAL MATERIAL

Recalibrating differential gene expression by genetic dosage variance prioritizes functionally relevant genes

Philipp Rentzsch, Aaron Kollotzek, Kaushik Ram Ganapathy, Pejman Mohammadi, Tuuli Lappalainen

Supplemental Table S1: Comparison of the Enrichment *p*-values and number of genes between nominal FC and recalibration. Based selection of the top 2,000 genes in the *Alasoo et al.* IFNG DE experiment.

Supplemental Table S2: Effect of tissue-specific recalibration. Comparison of the Enrichment *p*-values and number of genes between nominal FC, mean-recalibration and BRNCTXB-recalibration based selection of the top 700 genes in the psychENCODE ASD DE experiment.

Supplemental Table S3: Table of all V^G_H estimates. V^G_H as generated from GTEx v8 for 50 tissues and the weighted harmonic mean of all tissues.

Supplemental Table S4: Table of all V^G_{HI} estimates. V^G_{HI} and predictions from V^G_I are joined, with V^G_H taking precedence if both exist.

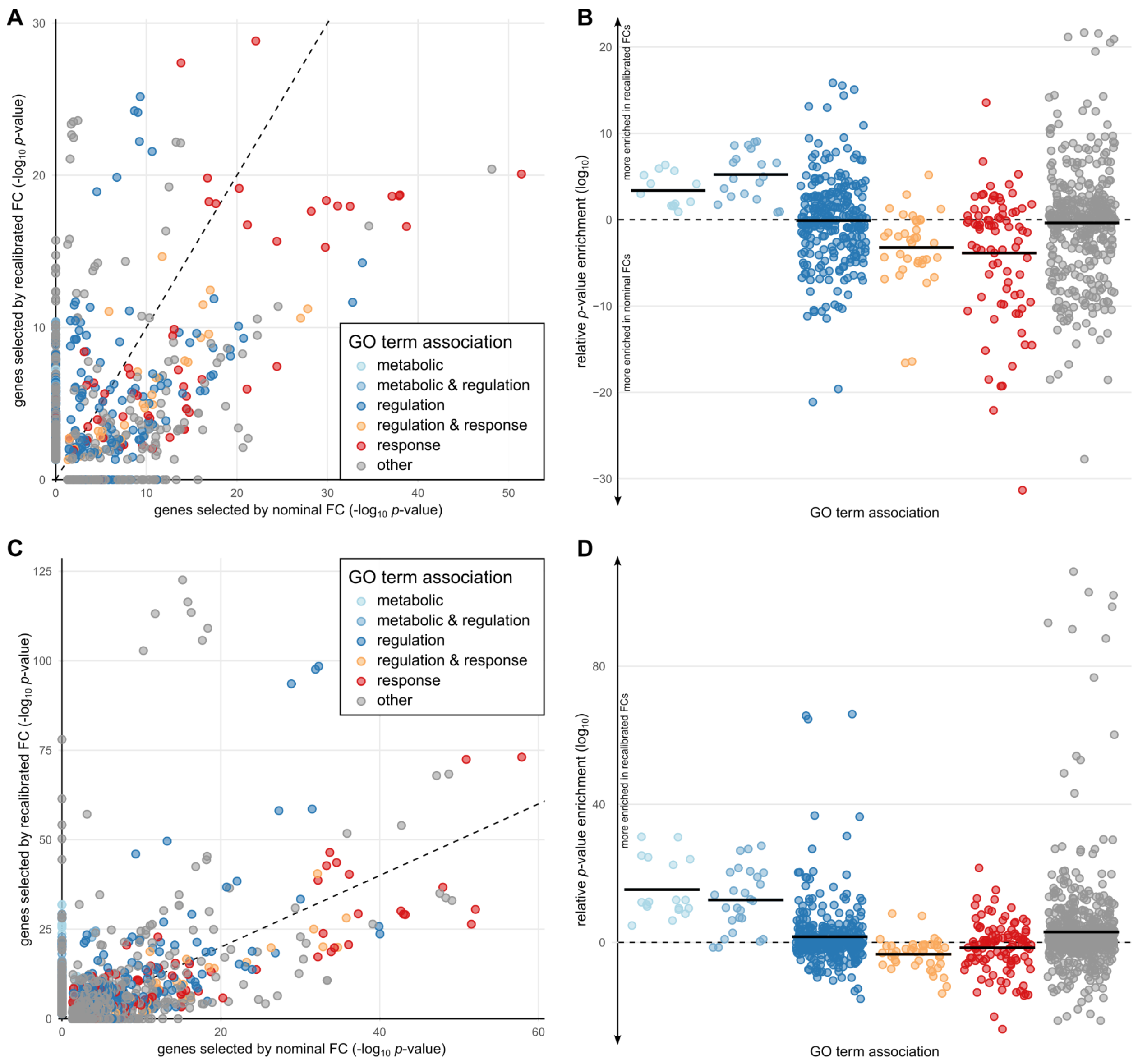

Supplemental Figure S1: GO term enrichment at different gene numbers. Equivalent figure to Fig. 2 E&F but restricting enrichment to the top 1,000 (**A&B**) and 4,000 (**C&D**) genes for GO term enrichment testing

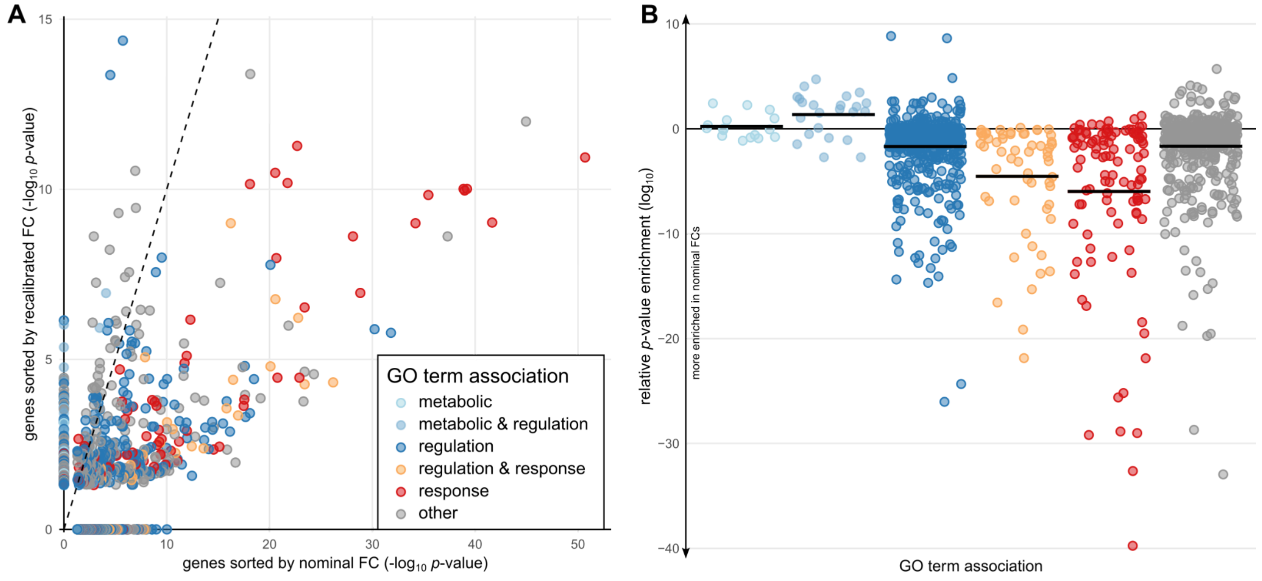

Supplemental Figure S2: Gene Set Enrichment analysis of all significantly DE genes. Similar to Fig. 2 E&F but based on ranking of genes (Gene Set Enrichment, GSE). **A)** GO term GSE *p*-values when genes are sorted by nominal fold change (y-axis) compared to recalibrated fold-changes (x-axis). **B)** Changes in the GSE *p*-values grouped by association.

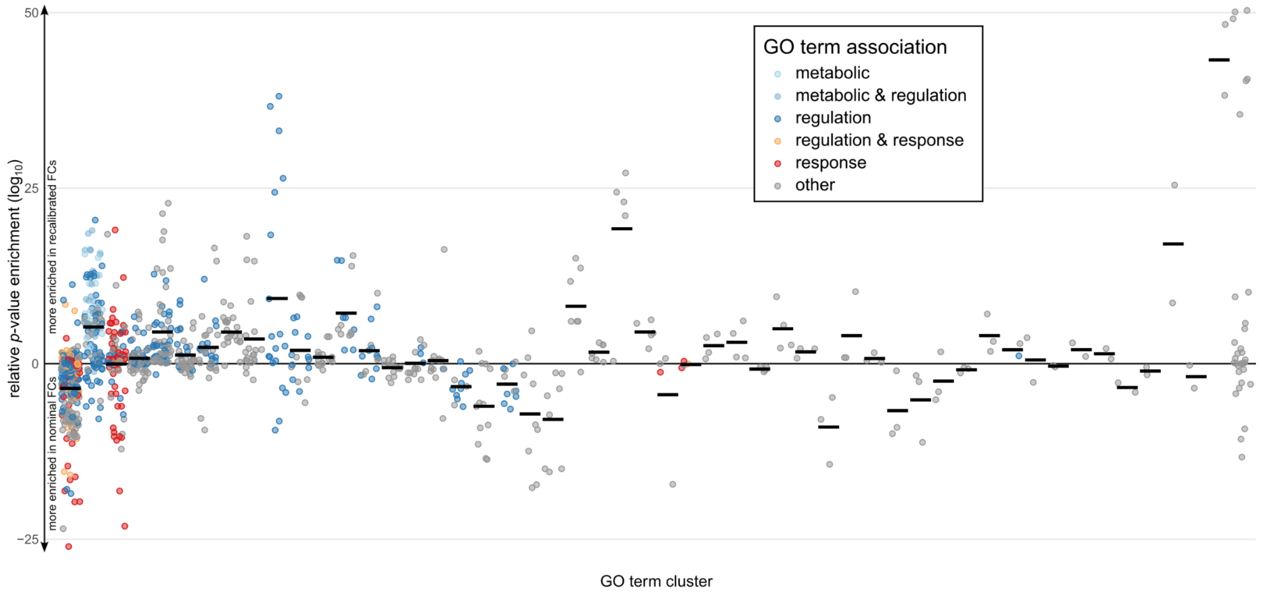

Supplemental Figure S3: Enrichment in GO term cluster. Difference in GO term enrichments equivalent to Fig 2F, but by GO cluster. Clusters consist of GO terms that contain similar genes. The rightmost column are 34 GO terms that do not cluster with any other term.

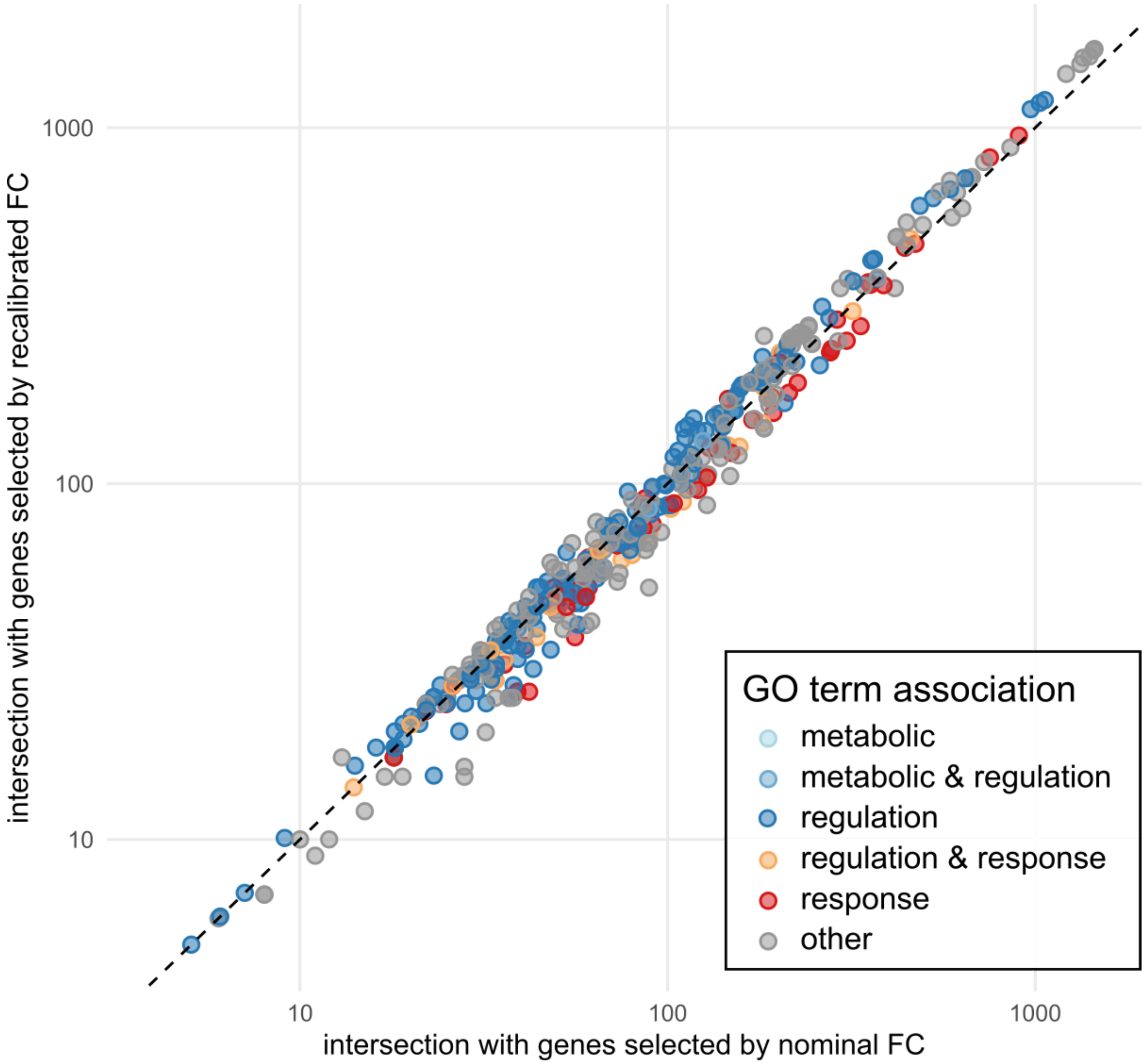

Supplemental Figure S4: **Absolute gene numbers matching per GO term before and after recalibration.** Based on selection of top 2,000 genes by absolute nominal and recalibrated fold change respectively.

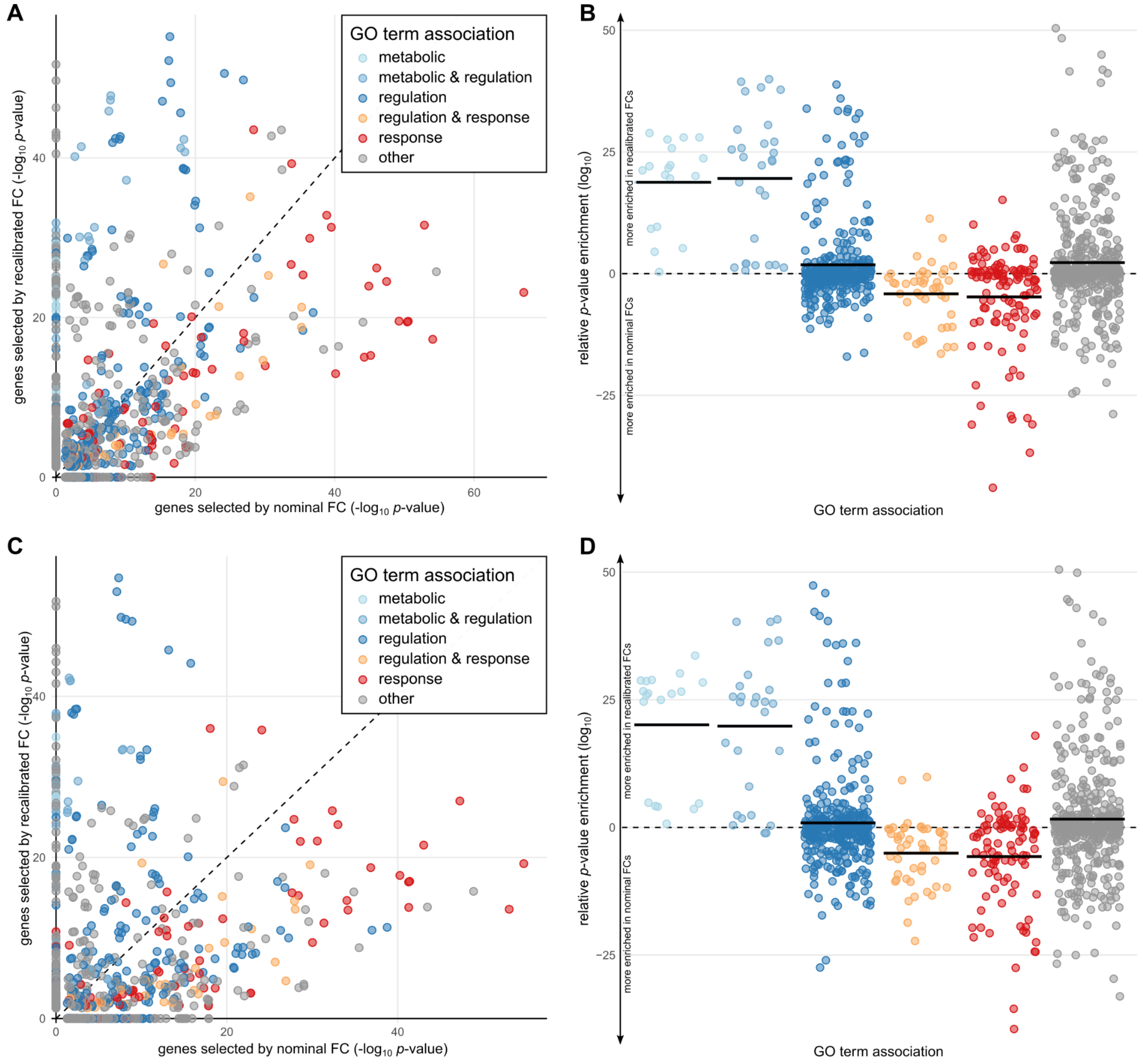

Supplemental Figure S5: **Other perturbations from the same experimental series.** Equivalent figures to Fig. 2E&F but from different experiments. **A&B**) Perturbation with *Salmonella*. **C&D**) Perturbation with IFNG and *Salmonella*.

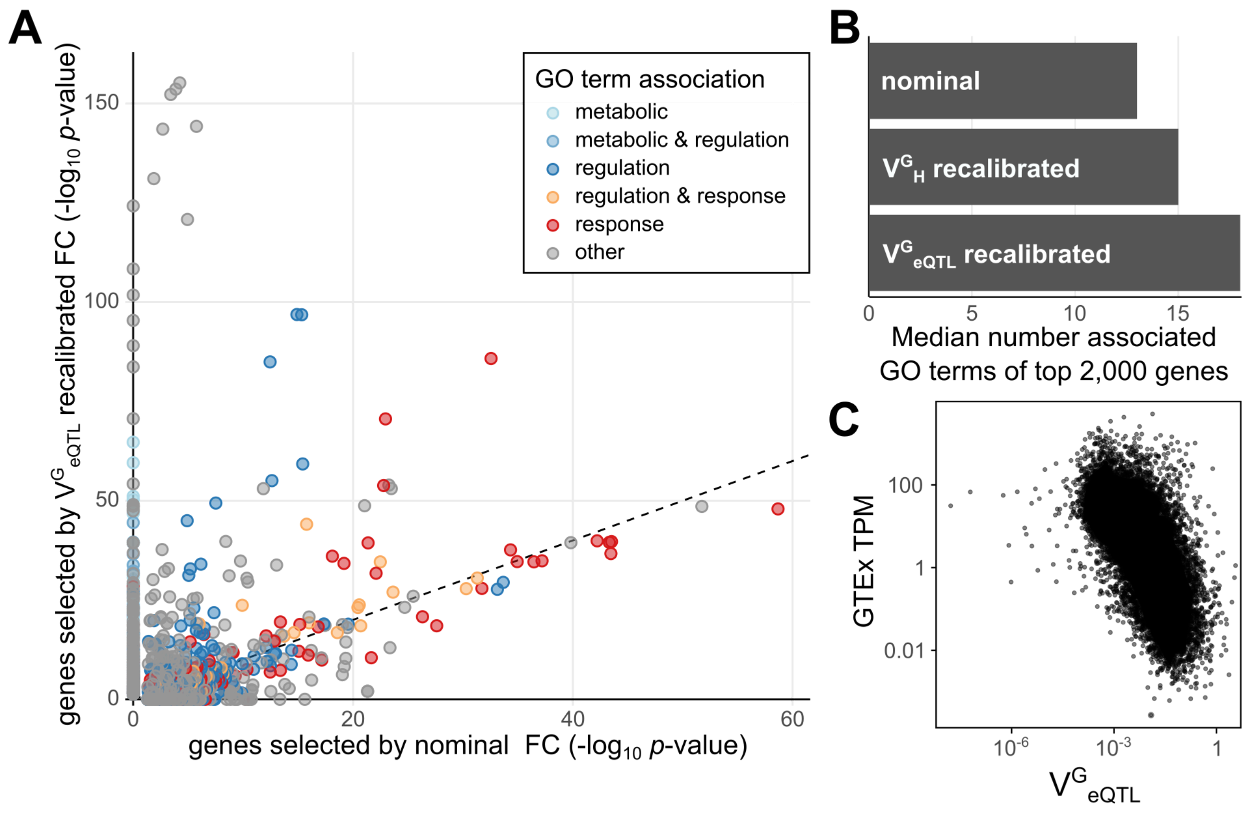

Supplemental Figure S6: Recalibration with eQTL-based V^G. A) Impact of recalibration on GO term enrichment in the IFNG stimulus dataset when using eQTL-derived V^G. eQTL-based V^G for recalibration resulted in a similar trend of raising regulatory over response GO terms comparable, with generally higher enrichment *p*-values due to the larger number of genes (**B**). This effect can be explained best by the higher number of GO terms associated with genes prioritized by recalibration with V^G_{eQTL}. The more significant *p*-values are hence likely the result of the number of GO terms being correlated with mean expression ($\rho_{Spearman's}$ = 0.65) and V^G_{eQTL} being strongly correlated with the GTEx mean TPM (**C**, $\rho_{Spearman's}$ = -0.76, compared to $\rho_{Spearman's}$ = -0.2834 for V^G_H). While some of this correlation is likely due to biology, with highly expressed genes being more constrained (Lek et al. 2016), the high correlation for V^G_{eQTL} may derive e.g. from differences in the power of eQTL calling. This, and the fact that allele-specificAE data is

naturally unaffected by environmental and technical factors, motivated the primary use of V^G_H for recalibration.

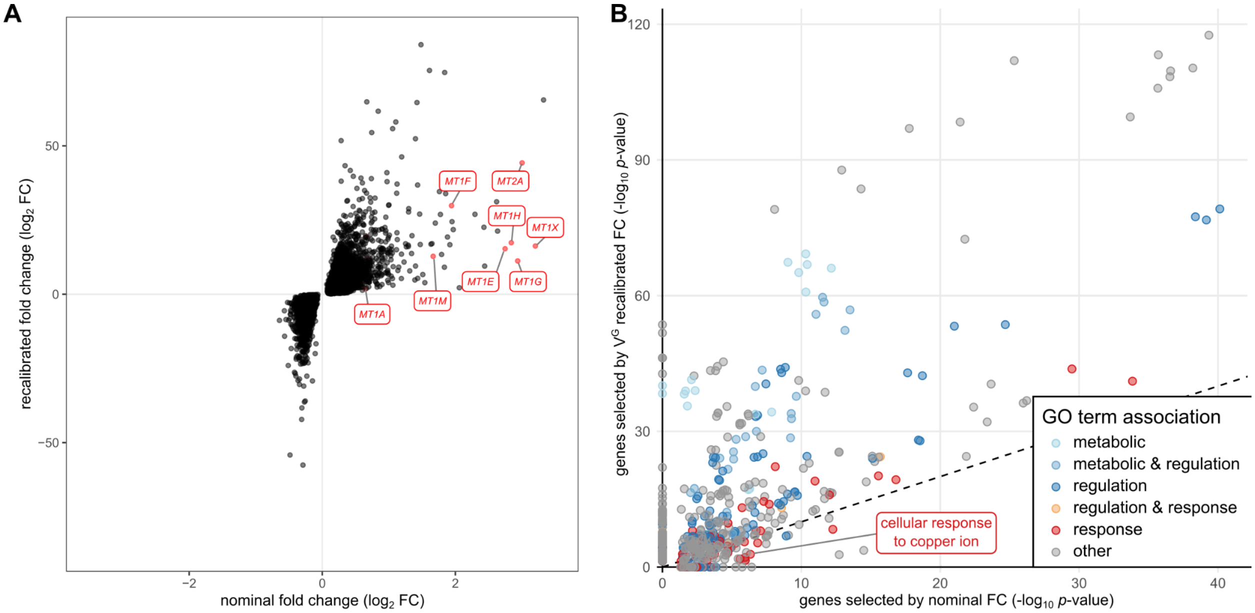

Supplemental Figure S7: Recalibration of experiment where IPSCs are perturbed with copper ion solution. A) Absolute nominal compared to recalibrated fold changes of the copper ion perturbation experiment. Highlighted in red are genes associated with the GO term ‘cellular response to copper ion’. Methallothionine genes (*MT*) are among the most down ranked genes after recalibration. **B)** GO term enrichment of DE experiment in the top 2,000 genes before and after recalibration.

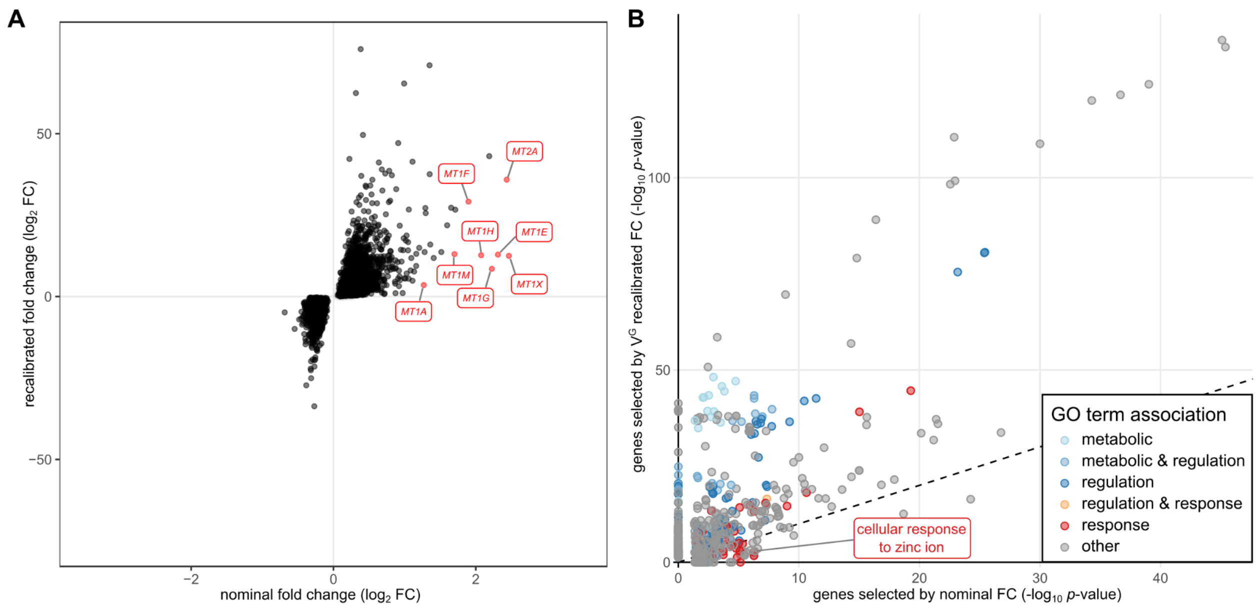

Supplemental Figure S8: Recalibration of experiment where IPSCs are perturbed with zinc ion solution. A) Absolute nominal compared to recalibrated fold changes of the zinc ion perturbation experiment. Highlighted in red are genes associated with the GO term ‘intracellular zinc ion homeostasis’. Methallothionine genes (*MT*) are among the most down ranked genes after recalibration. **B)** GO term enrichment of DE experiment in the top 2,000 genes before and after recalibration.

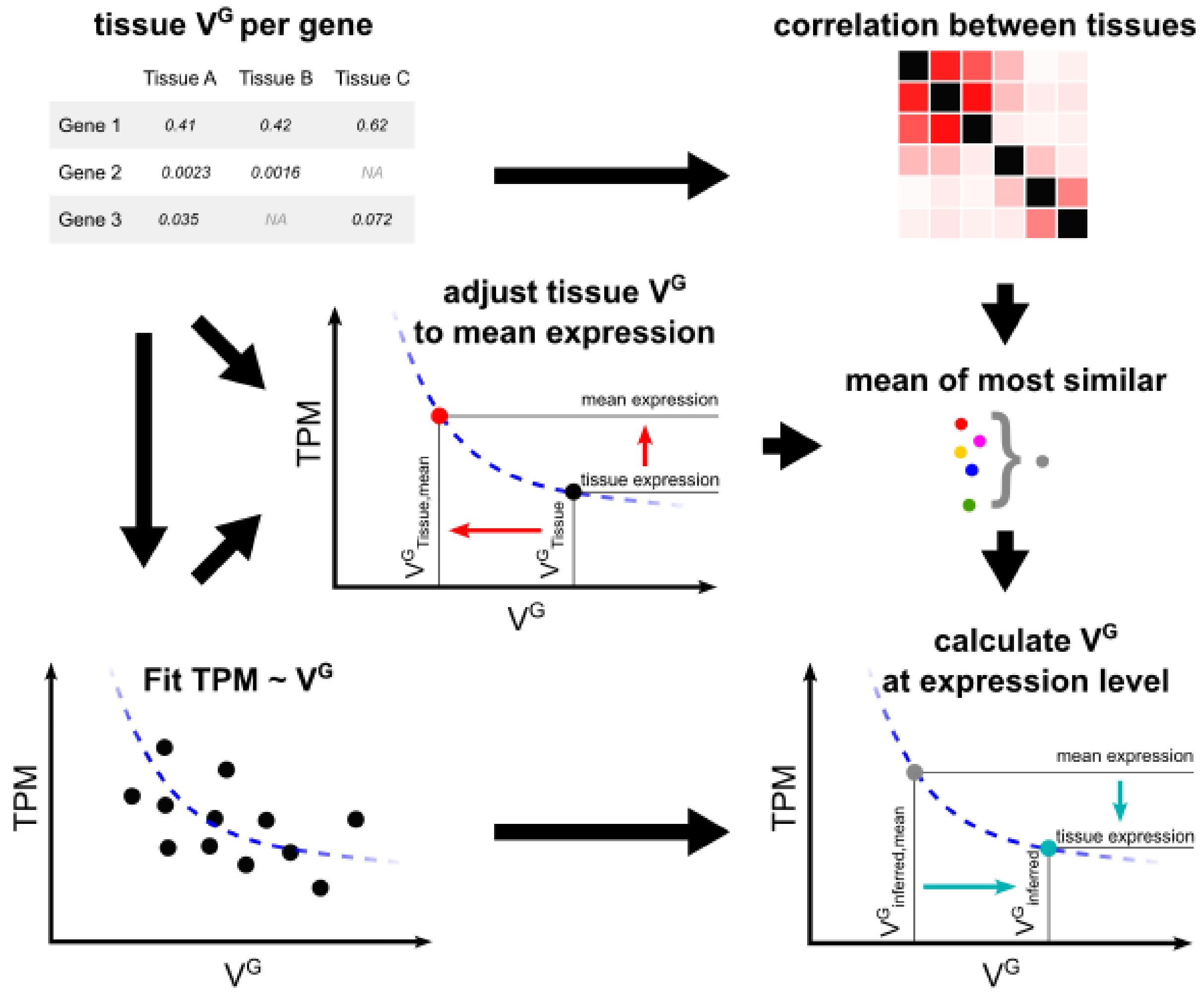

Supplemental Figure S9: Method outline to infer tissue-specific V^G estimates. The method enables the inference of V^G estimates based on similar tissues and adjusted for gene expression in each tissue.

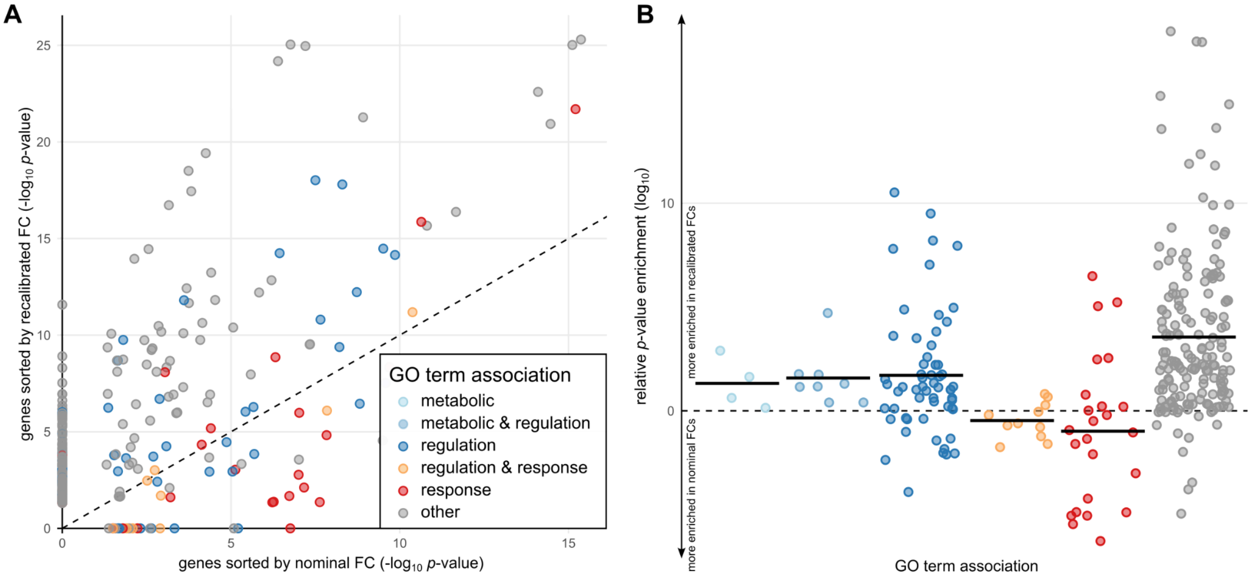

Supplemental Figure S10: Effect of tissue-specific recalibration. Plot similar to Fig. 2E&F but for the psychENCODE ASD experiment. Enrichment comparison of the top 700 genes selected by recalibration with $V^{G}_{HI,BRNCTXB}$ and nominal fold change.

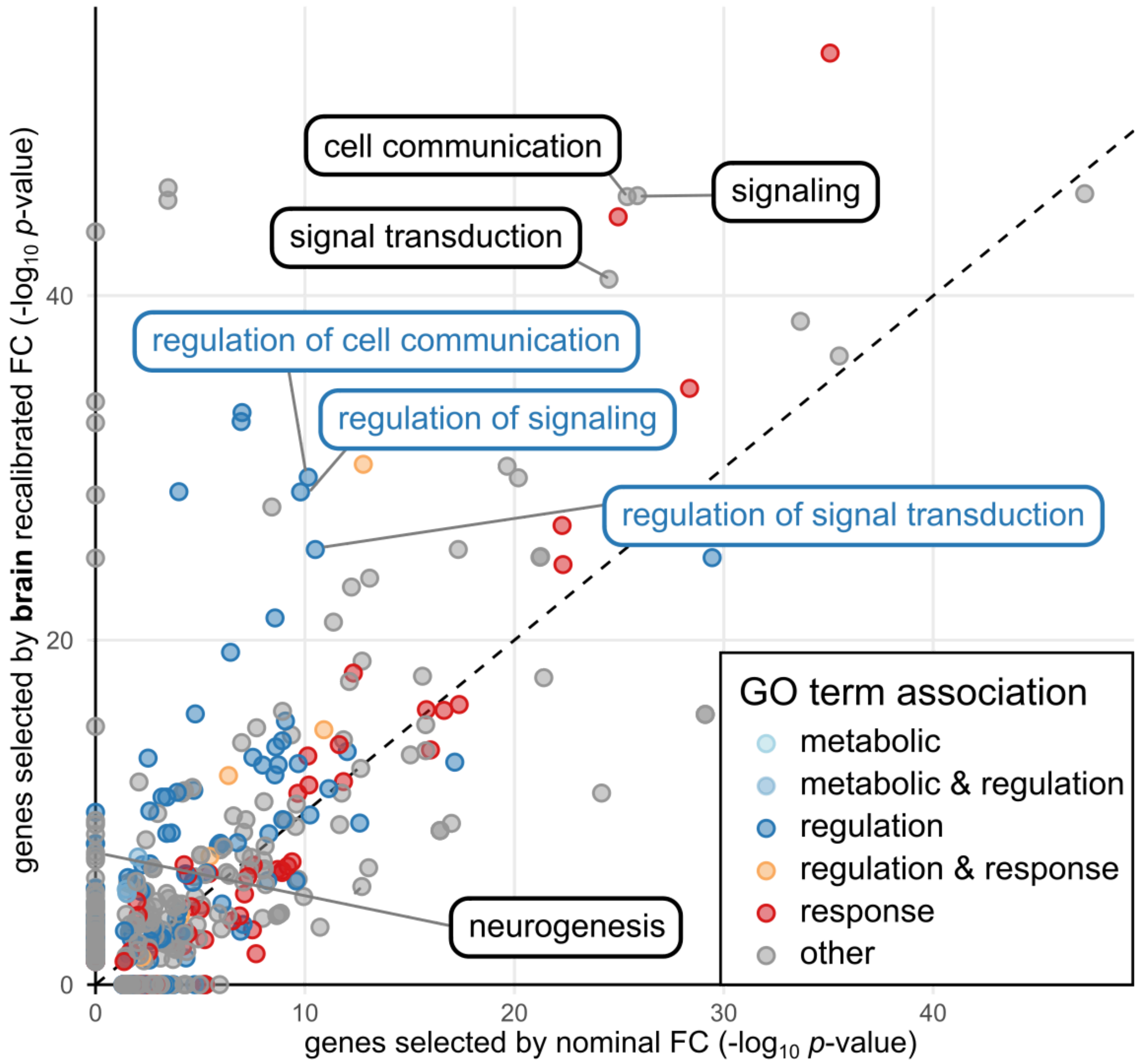

Supplemental Figure S11: GO term enrichments of top 2,000 genes from psychENCODE Schizophrenia DE experiment. Recalibrated with brain-specific V^G ($V^G_{HI,BRNCTXB}$). Some of the most important GO terms related to cell signaling and neurogenesis are highlighted.

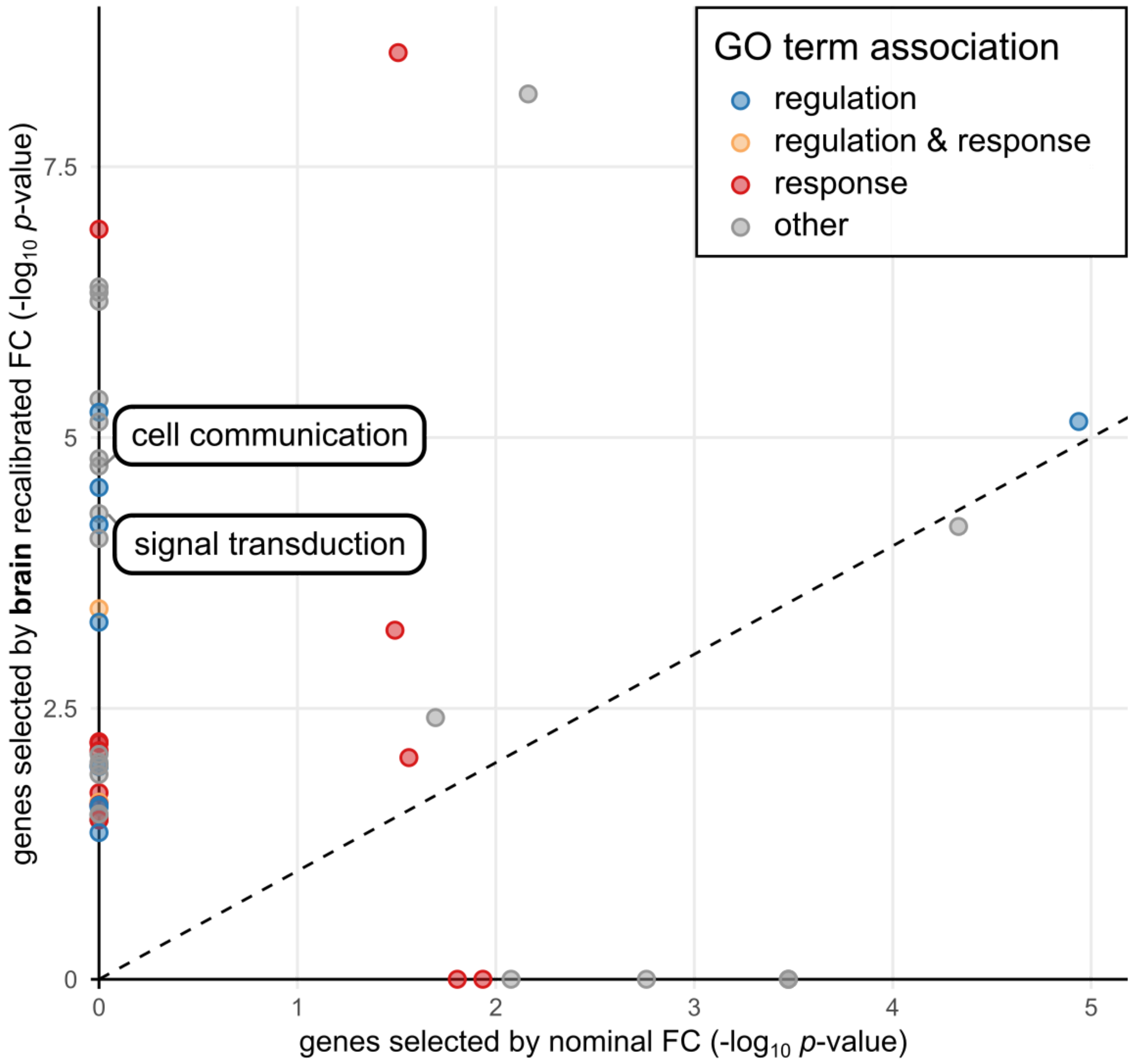

Supplemental Figure S12: GO term enrichments of top 500 genes from psychENCODE Bipolar Disorder DE experiment. Recalibrated with brain-specific V^G ($V^G_{HIAEML,BRNCTXB}$). Some of the most important GO terms related to cell signaling that only appear after recalibration are highlighted.

Supplemental Code S1: Implementation of recalibration in the R programming language.

```
#!/usr/bin/Rscript

# Load required packages
library(readxl)
library(utils)
library(ggplot2)
library(gprofiler2)

### Code to perform recalibration
#' @param df_de data.frame: contains differential expression per
gene. Row names
#' are Ensembl gene ids and one column contains the log fold change.
#' @param vg data.frame: contains the V^G estimate per gene (rows)
for one
#' or multiple tissues (columns). Default = [vg_h], the haplotype
expression
#' based V^G estimates calculated from GTEx v8.
#' @param tissue char: V^G tissue that is recalibrated against.
Default V^G are
#' generated for the GTEx tissues (in GTEx 6-letter code) or MEAN
(weighted
#' harmonic mean across tissues). Default = "MEAN".
#' @param remove_NA bool: whether genes for which no V^G estimate
exist should
#' be removed from the final data.frame. Default = FALSE.
#' @param sort_by char: sort result data.frame by one particular
column.
#' Default = NA
#' @param add_vg bool: adds the V^G estimates used for recalibration
to the
#' result data.frame. Default = FALSE.
#' @param variance_offset numeric: add an offset to all V^G
estimates. Default
#' = 0.
#' @param FC_col_name char: column of df_de that contains the log
fold change
#' values that are recalibrated. Default = "log2FoldChange".
#' @returns A modified data.frame with added recalibrated fold
changes.
#' @examples
#' df <- data.frame(
#'   log2FoldChange = c(-2.95, 1.03, 4.34),
#'   padj = c(0, 1e-2, 1e-5),
#'   row.names = c("ENSG00000000003", "ENSG00000000419",
"ENSG00000000457")
#' )
#'
```

```
#' recalibrateFoldChange(df)
#' recalibrateFoldChange(df, tissue = "NERVET", vg = vg_hi)
#' recalibrateFoldChange(df, sort_by = "padj", add_vg = TRUE)
#' @export
recalibrateFoldChange <- function(df_de, vg = "vg_h", tissue =
"MEAN", remove_NA = FALSE,
                                  sort_by = NA, add_vg = FALSE,
variance_offset = 0,
                                  FC_col_name = "log2FoldChange") {

  if (!is.element(tissue, colnames(vg))) {
    stop("Unknown tissue. You have to specify one GTEx tissue in
short GTEx notation or use 'MEAN'.")
  }

  # genes = row.names(vg)[which(!is.na(vg[,tissue]))]
  # vg_tissue = vg[which(!is.na(vg[,tissue])), tissue]

  vg_select <- vg[row.names(df_de), tissue]
  sdg_select <- sqrt(vg_select + variance_offset)

  df_de$recalibratedFC <- df_de[, FC_col_name] / sdg_select

  if (add_vg) {
    df_de$vg <- vg_select
  }

  if (remove_NA) {
    df_de <- df_de[which(!is.na(df_de$recalibratedFC)), ]
  }

  if (is.element(sort_by, colnames(df_de))) {
    df_de <- df_de[order(df_de[, sort_by]), ]
  }

  return(df_de)
}

### load V^G_H from supplementary table S3
file_tblS3 = 'Supplemental_Table_S3.xlsx'
vgh_tbl <- read_excel(file_tblS3, sheet = "Supplemental Table S3")
vgh <- as.data.frame(vgh_tbl)
rownames(vgh) <- vgh$gene_id
vgh$gene_id <- NULL
vgh[] <- lapply(vgh, function(x) {
  suppressWarnings(as.numeric(as.character(x)))
})

### Analysis example
# Download the file from the URL and save it to the temporary file
```

```
url <-
"https://zenodo.org/records/839011/files/naive_vs_IFNg_DESeq2_fold_ch
ange.txt.gz"
temp_file_path <- tempfile(fileext = ".txt.gz")
download.file(url, destfile = temp_file_path, mode = "wb")
df <- read.table(temp_file_path, header = TRUE,
                 row.names = "gene_id")
# Delete the temporary file
unlink(temp_file_path)

df <- recalibrateFoldChange(df, remove_NA = TRUE)
ggplot(data = df, aes(y = recalibratedFC, x = log2FoldChange)) +
  theme_bw() +
  geom_point(alpha = 0.5, size = 1.2) +
  ylab(bquote("recalibrated fold change (log FC /" ~
              sqrt(V^G) ~ ")")) +
  xlab("nominal fold change (log FC)")

### GO term enrichment comparison
top_xgenes <- 2000

# select a background set
bg_genelist <- intersect(rownames(df), rownames(vg_h))

# only select significant genes
df_sig <- subset(df, padj < 0.05)

# select top genes by nominal and recalibrated FC
nfc_order <- order(abs(df_sig$log2FoldChange), decreasing = T)
rfc_order <- order(abs(df_sig$recalibratedFC), decreasing = T)
nfc_genes <- rownames(df_sig)[nfc_order[1:top_xgenes]]
rfc_genes <- rownames(df_sig)[rfc_order[1:top_xgenes]]

# perform GO enrichment
enrichment <- function(gene.list, bglist) {
  gostres <- gost(
    query = gene.list, organism = "hsapiens", ordered_query = FALSE,
    multi_query = FALSE, significant = TRUE, exclude_iea = FALSE,
    measure_underrepresentation = FALSE, evcodes = FALSE,
    user_threshold = 0.05, correction_method = "g_SCS",
    domain_scope = "custom", custom_bg = bglist,
    numeric_ns = "", sources = "GO", as_short_link = FALSE
  )
  gores <- as.data.frame(gostres$result[, c(3, 11)])
  rownames(gores) <- gores$term_name
  return(gores)
}
gores <- enrichment(nfc_genes, bg_genelist)
rfc_gores <- enrichment(rfc_genes, bg_genelist)
```

```
# join enrichments
gores$p_value_rfc <- 1 # impute all non-hits from rfc
for (term in row.names(rfc_gores)) {
  if (!term %in% row.names(gores)) { # add missing GO terms to nfc
    gores[term, "term_name"] <- term
    gores[term, "p_value"] <- 1
  }
  gores[term, "p_value_rfc"] <- rfc_gores[term, "p_value"]
}
rownames(gores) <- gores$term_name

# match GO terms based on strings
termAssociations <- c("regulation", "response")
gores$association <- "other"
for (term in row.names(gores)) {
  for (match in termAssociations) {
    if (grepl(match, term, fixed = T)) {
      if (gores[term, "association"] == "other") {
        gores[term, "association"] <- match
      } else {
        gores[term, "association"] <- paste(
                      gores[term, "Association"], match, sep = " & ")
      }
    }
  }
}

# plot GO term associations by method of gene selection
plot.colors <- c(
  "regulation" = "#0000FF", "regulation & response" = "#990099",
  "response" = "#FF0000", "other" = "#999999"
)
ggplot(gores, aes(-log10(p_value), -log10(p_value_rfc),
                  color = association)) +
  geom_point() +
  geom_abline(intercept = 0, slope = 1, linetype = 2) +
  theme_minimal() +
  scale_color_manual(name = "GO Term association",
                     values = plot.colors) +
  scale_x_continuous(name = "selected by nominal FC" ~
                     -log[10] ~ "p-value") +
  scale_y_continuous(name = "selected by recalibrated FC" ~
                     -log[10] ~ "p-value") +
  ggtitle(paste("GO enrichment of the top", top_xgenes, "genes")) +
  theme(plot.title = element_text(hjust = 0.5, face = "bold"))
```

Supplemental Code S2: Inference of V^G for additional tissues in the R programming language.

```
#!/usr/bin/Rscript

# Load required packages
library(tidyverse)
library(data.table)
library(recalibration)

#' Load GTEx gene expression data from TPM file
#'
#' @param tpm_path Path to GTEx TPM file
#' @return Data frame containing tissue TPM values
load_gtex_data <- function(tpm_path) {
    # Read GTEx TPM file, skipping header rows
    tpm_data <- read.table(tpm_path, sep = "\t", skip = 2,
                           header = TRUE)

    # Filter genes with zero expression across all tissues
    expressed_genes <- apply(tpm_data[3:56], 1, sum) > 0
    tpm_data <- tpm_data[expressed_genes, ]

    # Clean Ensembl gene IDs by removing version numbers
    rownames(tpm_data) <- sapply(
        tpm_data$Name,
        function(x) strsplit(x, "\\.")[[1]][1]
    )

    return(tpm_data)
}

#' Process GTEx colors information
#'
#' @param color_path Path to GTEx colors file
#' @return Data frame containing tissue color information
process_gtex_colors <- function(color_path) {
    gtex_infodf <- read.table(
        color_path,
        sep = "\t",
        header = TRUE,
        row.names = 'tissue_id'
    )

    # Add Kidney Medulla entry
    gtex_infodf['Kidney_Medulla', ] <- c('Kidney - Medulla',
                                          'KDNMED', '', '')
```

```
    # Convert tissue site detail to dot-string format
    gtex_infodf$dotstring <- sapply(
            gtex_infodf$tissue_site_detail,
            function(s) gsub("[-(), ]", ".", s)
    )
    rownames(gtex_infodf) <- gtex_infodf$dotstring

    return(gtex_infodf)
}

#' Calculate pseudocount-adjusted TPM values
#'
#' @param tpm_data Input TPM data
#' @return Data frame with adjusted TPM values
calculate_pseudocount_tpm <- function(tpm_data) {
    # Find minimum positive TPM value per tissue
    smallest_tissuetpm <- sapply(colnames(tpm_data)[3:56],
        function(i) {min(tpm_data[, i][tpm_data[, i] > 0])})

    # Convert to numeric and add pseudocount
    tpm_adj <- as.data.frame(sapply(tpm_data[, 3:56], as.numeric))
    rownames(tpm_adj) <- rownames(tpm_data)
    tpm_adj <- sweep(tpm_adj, 2, smallest_tissuetpm, "+")

    return(list(
        adjusted_tpm = tpm_adj,
        mean_tpm = apply(tpm_data[, 3:56], 1, mean)
    ))
}

#' Adjust VG estimates based on TPM values
#'
#' @param vgh_df VG data frame
#' @param tissue_tpm TPM data frame
#' @param mean_tpm Mean TPM values
#' @return Adjusted VG data frame
adjust_vg_estimates <- function(vgh_df, tissue_tpm, mean_tpm) {
    # Filter genes present in both datasets
    vgh_df_adj <- vgh_df[rownames(vgh_df) %in% rownames(tissue_tpm),]
    vgh_df_adj <- vgh_df_adj[!is.na(apply(vgh_df_adj,
                FUN = function(x) {mean(x, na.rm = TRUE)}, 1)), ]

    tissues <- colnames(tissue_tpm)
    tissues <- tissues[tissues %in% colnames(vgh_df)]

    for (tissue in tissues) {
        df_tissue <- data.frame(
            row.names = rownames(vgh_df),
            vg = vgh_df[, tissue],
            tpm = tissue_tpm[rownames(vgh_df), tissue]
```

```
        )

        df_tissue <- subset(df_tissue, vg > 0 & tpm > 0)
        genes <- rownames(df_tissue)

        model <- lm(log10(vg) ~ log10(tpm), data = df_tissue)
        df_tissue$vg_adj <- (mean_tpm[genes] / df_tissue$tpm) \
           ** model$coefficients[2] * df_tissue$vg

        vgh_df_adj[genes, tissue] <- df_tissue$vg_adj
    }

    return(vgh_df_adj)
}

#' Calculate weighted VG values based on correlation
#'
#' @param gene Gene symbol
#' @param weight Weight vector
#' @param correlations Correlation matrix
#' @param n Number of top correlations to consider
#' @return Weighted VG estimates
calculate_weighted_vg <- function(gene, weight, correlations, n = 5)
{
    # Filter non-NA weights
    weight <- weight[names(weight)[!is.na(weight)]]
    vgvalues <- vgh_df_adj[gene, names(weight)]

    # Filter tissues with VG values
    vgvalues <- vgvalues[names(vgvalues)[!is.na(vgvalues)]]
    weight <- weight[names(vgvalues)]

    # Select top n correlations if available
    if (length(weight) > n) {
        correlations <- correlations[names(vgvalues)]
        correlations <- correlations[order(correlations,
                                  decreasing = TRUE)[1:n]]
        vgvalues <- vgvalues[names(correlations)]
        weight <- weight[names(correlations)]
    }

    return(sum(weight * vgvalues) / sum(weight))
}

# Main analysis pipeline
main() {
    # Load GTEx expression data. File from gtexportal.org
    tpm_data <-load_gtex_data(
'GTEx_Analysis_2017-06-05_v8_RNASeQCv1.1.9_gene_median_tpm.gct.gz')
```

```
    # Use GTEx colors file to match TPM and VG tissue names. From:
    #
github.com/stephenslab/gtexresults/blob/master/data/GTExColors.txt
    gtex_infodf <- process_gtex_colors('gtex_colors.txt')
    colnames(tpm_data)[3:56] <- \
             gtex_infodf[colnames(tpm_data)[3:56], 'tissue_abbrv']

    # Calculate adjusted TPM values (with pseudocount)
    tpm_results <- calculate_pseudocount_tpm(tpm_data)
    tissue_tpm_adj <- tpm_results$adjusted_tpm
    mean_tpm <- tpm_results$mean_tpm

    # Adjust VG estimates to mean
    vgh_df_adj <- adjust_vg_estimates(vgh_df, tissue_tpm_adj,
                                      mean_tpm)

    # Calculate tissue correlations
    tissue_cor <- cor(vgh_df, method = 'spearman',
                      use = 'pairwise.complete.obs')

    # Create imputed VG estimates
    tissues <- colnames(tissue_tpm_adj)
    tissues <- tissues[tissues %in% colnames(vgh_df)]
    vgh_df_impute <- vgh_df_adj

    for (tissue in tissues) {
        # Get correlations excluding current tissue
        tissuesCorrelations <- tissue_cor[tissue,
               !colnames(tissue_cor) %in% c(tissue, 'MEAN')]

        # Calculate weights based on TPM and correlation
        tissueWeights <- as.data.frame(tissue_tpm_adj[,
          names(tissuesCorrelations)] ** 0 * tissuesCorrelations)

        # Impute VG estimates at mean expression
        vgh_df_impute[rownames(tissueWeights), tissue] <- sapply(
            rownames(tissueWeights),
            function(gene) calculate_weighted_vg(gene,
                   tissueWeights[gene, ], tissuesCorrelations)
        )

        # Readjust from mean TPM to tissue expression
        df_tissue <- data.frame(
            row.names = rownames(vgh_df),
            vg = vgh_df[, tissue],
            tpm = tissue_tpm_adj[rownames(vgh_df), tissue]
        )
        genes <- rownames(df_tissue)
        model <- lm(log10(vg) ~ log10(tpm),
                 data = subset(df_tissue, vg > 0 & tpm > 0))
```

```
        df_tissue$vg_adj <- (df_tissue$tpm/mean_tpm[genes]) \
          ** model$coefficients[2] * vgh_df_impute[genes, tissue]
        vgh_df_impute[genes, tissue] <- df_tissue$vg_adj
    }

    # Save results
    write.table(vgh_df_impute, file = 'VGHimputed.tsv',
                quote = FALSE, sep = '\t')
}

# Run main analysis
main()
```
